# Scenario on Production, Processing, and Utilization of Grasspea (*Lathyrus sativus* L.) in Agromarginal Geographies and Its Future Prospects

**DOI:** 10.1155/2024/8247993

**Published:** 2024-09-04

**Authors:** Lamesgen Yegrem, Asnake Fikre, Shashitu Alelign

**Affiliations:** Ethiopian Institute of Agricultural Research Deber Zeit Agricultural Research Center, Deber Zeit, Ethiopia

**Keywords:** *β*-ODAP, grasspea, nutritional composition and food processing

## Abstract

Grasspeas are environmentally successful and robust legumes with major traits of interest for food and nutrition security. It is a critical crop in areas prone to drought, overmoisture stress, and famine, hence, regarded as an “insurance crop” because of its inherent resilience of climatic calamities. The current status and prospects of grasspea, as well as various breeding and food processing approaches to improve this crop for integration in diverse and sustainable agrifood systems, are discussed in this review. Grasspeas are often the source of important micronutrients and proteins (18%–34%), saving peoples' lives during famine. Grasspea consumption is increasing in some countries; however, uninterrupted consumption of grasspea should be avoided, especially when they are green or unripe and when they are raw. Effective food processing techniques are essential to reduce the neurotoxic hazards associated with eating grasspea. Several effective processing steps can be used to reduce toxicity in addition to the development of toxin-free varieties for production and consumption. With advances in the scientific investigation of the grasspea, integration of genetics, processing, and behavioral components has been suggested.

## 1. Introduction

Grasspea (*Lathyrus sativus* L.) is a Neolithic plant that has been cultivated for millennia and has spread across three continents (Asia, Europe, and Africa). Many tropical and subtropical regions of the world, including Iraq, Iran, Afghanistan, Syria, Lebanon, India, Pakistan, Bangladesh, Ethiopia, Algeria, Egypt, Libya, Morocco, France, Spain, North America, and temperate South America, commonly produce and use grasspea. Indian vetch (United Kingdom and North America), *Almorta* (Spain), *Khesari* or *Batura* (India), *Alverjas* (Venezuela), *Gilban* (Sudan), *Guaya* (Ethiopia), *Matri* (Pakistan), *Gesette* (France), and *Pisello bretonne* (Italy) are some of the local names used for crops throughout the world. Grasspeas were domesticated in the geographies along grain legumes such as peas and lentils, which were brought there from the Near East approximately 6000 BC. As a result of the spread of agriculture from the Near East, grasspea is likely among the earliest crops domesticated in Europe [1].

Using more underutilized crops that already demonstrate advantageous features in terms of tolerance to drought and other environmental stress factors is a supplement approach to the continuous development of key crop species to ensure sustainable food and feed production in the face of climate change. Grasspea can survive salt, some fungi, insect herbivores, droughts, and floods [2]. Owing to these characteristics, it has been able to thrive in agricultural conditions that are unsuitable for other common crops. It is grown under low-input agriculture, which at times greatly enhances the food security of disadvantaged smallholder farmers. When other crops fail due to adverse weather conditions, they can become the only source of food for the poorer segment of the population and occasionally act as an emergency food source. A nitrogen fixation rate of 124 kg/ha/year has been recorded [3], making grasspea a highly efficient crop for increasing soil nitrogen.

Grasspeas are essential for the provision of calories, proteins, and nutritional minerals [4]. It may be used for a variety of purposes, including human food, animal feed, fodder [5], and soil replenishment. However, like other legumes, grasspea seeds also contain a variety of antinutritional components (ANFs), particularly a free amino acid known as hazardous *β*-ODAP, which is found to have health hazards. Foods made from grasspea seeds were delicious. In several European nations (Spain, France, Portugal, Italy, and Poland), Africa (Ethiopia), and South Asia (India, Bangladesh, and Nepal), they are still popular food sources [6, 7]. Its consumption in Ethiopia includes green peas (*Eshet*), snack boiled whole seeds (*nifro*), roasted whole seeds (*kollo*), traditional sauces (*shiro-wot* and *kik wott*), local beverages (*areke*), and green unripe seeds (*eshet*) [3]. The staple *Injera*, a fermented, sour pancake-like leavened bread made of *Tef*, wheat, barley, maize, or sorghum, is being served, among others, with grasspea curry or *shiro-wot*.

### 1.1. Grasspea Production

Grasspeas are currently grown and naturalized in many regions of southern, central, and eastern Europe, as well as in the Mediterranean Basin, Iraq, and Afghanistan. It is a valuable crop in Bangladesh, India, Pakistan, Nepal, and Ethiopia [8]. In temperate regions, grasspea are spring crops; in subtropical regions, they are winter crops. It may grow everywhere with an average temperature between 10°C and 25°C, including at sea level up to 1200 m in India and 1700–2700 m in Ethiopia. It can be cultivated in regions with annual rainfall ranging from 400 to 650 mm. It can withstand heavy rains during the early stages of growth and prolonged drought. Grasspea thrives in a wide range of soil conditions, including poor soils, heavy clays, and resistance to waterlogging, as well as moderate alkalinity and salinity [9].

At 384,800 t, India is the top producer, followed by Bangladesh (232,500 t), and Ethiopia (202,126 t) [10]. Several countries, including Australia, Spain, Italy, and Canada, are working to expand their production as a break crop between cereals and as a bonus crop in fallow land because of their biological nitrogen-fixing qualities [11] for both animal feed and human food. Ethiopia is not only the continent's largest producer and consumer of grasspea (guaya), with an 85% market share. It is not only the plant's environmental tolerance but also major factors such as poverty and civil conflict that force people to rely on grasspea. Thus, Ethiopians are also the most frequent victims of toxicity [12], but it is not only the country's environmental tolerance but also major factors such as poverty and civil conflict that force people to rely on grasspea. The Ethiopian Biodiversity Institute collection has 578 accessions of grasspea, also known as *guaya* in Amharic. Grasspea is the fifth legume in terms of production and geographic reach behind chickpeas, haricot beans, field peas, and fava beans (Table 1) [13]. According to FAO statistics on production from 1996 to 2007, grasspea accounted for 75.3% and 9% of the area covered in Ethiopia, and 85.4% and 8% of the production from Africa and the rest of the world, respectively. It is primarily grown in Cambisol and Vertisol soils. In Ethiopia, West Tigray, South Tigray, East Tigray, and North Gonder, South Gonder, North Welo, South Welo, East Gojam, West Gojam, North Shewa, and East Shewa are the major producer–consumer administrative zones. It may also be found in Arsi, Bale, Hararge, and the Southern Regional State [3]. However, its production is more widespread in the northwestern (58%), central (16%), and northeastern (13%) regions of Brazil. The remaining 13% of the country's grasspea acreage is distributed over the northern and southern sections of the country [14].

Despite the extensive growth of grasspea in Ethiopians, only one low-*β*-ODAP variety (Wasie) has been released for production [3], which has, however, not been tested for potential neurotoxicity in an appropriate animal species. This kind was created through cooperation between the International Center for Agricultural Research in the Dry Regions (ICARDA) and the Ethiopian Institute of Agricultural Research (EIAR). With the aim of improving agronomic features and stability of low *β*-ODAP content to enable the generation of future improved low-*β*-ODAP cultivars, EIAR is now undertaking multilocation trials on several accessions.

## 2. Adaptability and Resilience Power of Grasspea in the Unpredicted Climatic Variables

According to predictions, by 2050, the world's food consumption will double owing to population and wealth development, which would result in more rivalry for crops used as sources of bioenergy, fiber, and other industrial sources (http://www.fao.org). Despite unfavorable growth conditions, grasspea is a hardy crop with reported endurance to severe temperatures, drought, flooding, and salinity. It can also grow successfully in warm regions and marginal or nutrient-deficient soils [15]. Water deficiency is one of the main factors that limit crop development and output. Water can be conserved by increasing a plant's water use efficiency and creating morphological drought tolerance features as adaptive mechanisms [16, 17]. These mechanisms correspond to three drought adaptation strategies: escape (crops complete their life cycle before the onset of terminal drought), avoidance (crops maximize water uptake while minimizing water loss), and tolerance (crops continue to grow and function at low water content) [18]. A drought avoidance technique is used by grasspea with winged and narrow leaves that can roll inward from leaf margins to reduce water loss [19]. Enhancing water collection efficiency through a deep root system, which provides access to untapped water resources when the soil surface desiccates, is another strategy for increasing the resistance of plants to the effects of climate change [20]. Grasspea, in fact, has a robust and penetrating root system, and it has a large number of proteins responsive to abiotic and biotic stressors [21], making it suitable for a variety of soil types [22].

Grasspea is viewed as an “insurance crop” that may flourish in marginal places, giving poor farmers stability in their economic, social, and dietary circumstances [23]. For more than 100 million people residing in Asia and Africa that regularly face droughts, grasspea serves as their primary source of energy. It is a highly adaptable crop that thrives locally and can withstand harsh weather conditions, including cold and hot waves, submersions, and heavy rains [19]. In addition, if there is a lack of nutrients or a buildup of heavy metals, they may be able to live in saline soil and other harsh climate conditions [24].

Grasspea grows in a range of locations with annual precipitation ranging from 300 to 1500 mm and is naturally able to withstand temperature changes. It can withstand drought, severe rain, and floods with an extraordinary fortitude. It thrives in environments between 10°C and 25°C [25]. Grasspea grows well in almost any kind of loam to clay loam soil and is best for growing grasspea. It is a hardy crop that grows well in dry climates and produces great seed harvest on poor soils. Grasspea farming typically occurs in heavy clay soils. Black, very retentive soils are suitable for grasspea cultivation. Because lime is sensitive to acidity, lime treatment [26] and its robust and deep roots can withstand a variety of soil conditions, including thick clay and extremely poor soil. It is resistant to several diseases and pests.

Grasspea is a resilient, hardy, and climate-smart legume crop that can be grown with little assistance from outside sources and endure salt, drought, and waterlogging. It is a multipurpose crop that produces grain, feed, vegetables, and straw, while increasing soil fertility through atmospheric nitrogen fixation. Its potential for cultivation in areas where other field crops cannot be grown because of poor soil quality and water constraints gives resource-poor farmers and consumers an exceptional opportunity for sustainable agriculture and nutritional security (https://www.icarda.org/research/climate-smart-crops/grass-pea).

### 2.1. Is Grasspea Safe to Use?

Grasspeas have drawn both praise and criticism over their long history as a crop. Grasspeas were a component of the burial offerings discovered in the pyramids during the Pharaonic era [27]. It was discovered that consuming too many grasspea seeds could cause lathyrism, a debilitating neurological condition. Grasspea is safe to eat in small amounts. Nevertheless, consuming it as a main component of one's diet for 3 months can result in lifelong paralysis of legs bilaterally in adults and children through degeneration of the CNS, a condition known as lathyrism. The presence of *β*-ODAP, which causes neurological lathyrism in humans, is a significant obstacle to its production. Despite the presence of *β*-ODAP, the crop has recently gained prominence as a nutritious meal that promotes health. The Consultative Group on International Agricultural Research (CGIAR) estimates that at least 100,000 people in underdeveloped nations are paralyzed as a result of neurotoxicity.

Several communications emphasized childhood as a high-risk group [28], and some 7% of the 77 patients seen at the MNI in Barcelona were children under the age of 15 [29]. The youngest child recorded was a 27-month-old girl who was unable to walk after being fed grasspea for 3 months, dying shortly thereafter [30]. In one family, three male siblings, aged 5, 8, and 9 years, became ill simultaneously [31]. The clinical picture was similar to that of adults, except for some peculiarities. A dramatic case was a child who developed lathyrism after being fed grasspea in just 15 meals, while all children reportedly had been fed with grasspea porridge for a couple of months at most [32]. Lathyrism in infants has been scarcely addressed in the literature, despite the fact that its prevalence has reached 25% of patients in some series [33]. Most children usually developed severe forms of the disease following very short dietary exposures to quantities of grasspea not exceeding 100 g per day. According to a study by Dwivedi, Kumar, and Tripathi [6], lathyrism in infants can result in a range of motor deficits, including spasticity, muscle weakness, and permanent paralysis. These symptoms arise due to the excitotoxic effects of *β*-ODAP on motor neurons in the spinal cord, which are particularly vulnerable during early development.

The neurotoxic substance *β*-ODAP, which is present in grasspea seeds, is suspected to be the cause of the lathyrism. Up to 5% of the population may develop lathyrism when constant dietary ratios, including at least 25% grasspea, are consumed for 1.5–6 months. Lathyrism outbreaks are frequent in situations where people rely too heavily on grasspea. Grasspea seeds have a *β*-ODAP content of 0.1–1.4 g per 100 g seed [34].

Meanwhile, the underlying molecular causes of the neurodegenerative condition known as lathyrism, which can only be caused by consuming many grasspea seeds over an extended period of time, have been identified [35]. When grasspea are consumed as part of a diversified diet, *β*-ODAP is tolerated without any known negative effects [34]. There are several techniques to make grasspea less dangerous, such as washing and soaking the grasspea and then discarding the water before cooking, or eating grasspea combined with other crops. Both techniques are efficient in lowering the danger of lathyrism; however, detoxification of grasspea may be more difficult during famine when water and other food supplies are scarce. The quantity of the pulse ingested, the *β*-ODAP level, the cooking method, and the person's nutritional state all affect the incidence and probability of lathyrism outcomes. Even within a family, susceptibility to lathyrism differs among individuals. Additionally, agronomic studies have determined that *β*-ODAP content can be decreased by altering cultivation conditions. Hence, the composition of the crop, which is high in proteins, starch, vitamins (group B vitamins), fibers, calcium, and phosphorous, could be a new opportunity [34].

### 2.2. Grasspea as Functional Food

Legumes have great potential as antioxidants and can be lucrative crops [36]. Several techniques have been used to examine the antioxidant and antiradical properties of leguminous seed extracts, including liposomes, enhanced chemiluminescence, a model system based on carotene and linoleate, reducing power assay, LDL cholesterol oxidation, ferric-reducing antioxidant power (FRAP) assay, Fe^2+^-chelating capacity assay, and hydrophilic oxyglycan assay [37]. The total phenolic content of grasspea flour was estimated to be between 0.22 and 0.27 g/100 g [38]. Using DPPH, FRAP, and carotene bleaching techniques, the phenolic content of grasspea extracts was associated with their antioxidant activities. For grasspea extracts, Menga, Codianni, and Fares [39] found linear relationships between the quantity of total phenolics, total flavonoids, and condensed tannins and the outcomes of the ABTS assay.

A balanced diet and moderate consumption may be beneficial. Grasspea seeds have the potential to cause motor neuron degeneration when consumed regularly for an extended period in specific socioeconomic contexts, as it would be the most affordable dietary option. It has been demonstrated that *β*-ODAP has hemostatic properties that seem to result from vasoconstrictive action rather than any effect on blood coagulation [40]. In order to profit from this bioactive property, Yunnan Baiyao (Kunming, Yunnan Province) is creating Band-Aids that contain *β*-ODAP to limit blood flow [41]. In addition, *β*-ODAP has been investigated for its bioactive properties, which may be beneficial in the treatment of hypoxia [42] and Alzheimer's disease [43], and it has recently been patented as a drug for the treatment of thrombocytopenia [44].

One kilogram of grasspea seeds every day, as Bangladeshi farmers use to say, gives them energy to labor in the fields. It is possible that the seed's 1% homoarginine content increases stamina [45]. Supplements for sports and bodybuilding include homoarginine. Homoarginine is a substitute for the protein amino acid arginine as a substrate for nitric oxide (NO) synthase. NO is a hormone-like metabolite that contributes to oxidative stress and several physiological procreesses, including nerve signal transmission and vasodilation during erection. NO is a volatile and short-lived molecule. According to recent research, low circulating homoarginine levels are a risk factor for stroke and vascular disorders.

The tolerance threshold might thus be as high as 2 g of *β*-ODAP intake per day if just 0.5 kg of grasspea is consumed daily along with grains [19]. Lathyrism was dramatically reduced by adding gravel seasoned with antioxidant-rich spices to the plate [46]. The activation of protein kinase C, one of the physiological actions of *β*-ODAP with potential therapeutic value, provides a fresh perspective for investigating the potential of grasspea in the treatment of Alzheimer's disease, hypoxia, and long-term potentiation of neurons crucial for memory [47]. It is claimed that, however, low doses of *β*-ODAP are neuroprotective. It has been claimed that this amino acid, also known as dencichine and present in various *Panax* species, is a neuroexcitatory amino acid with antihemorrhagic properties. Hence, this “neuro-excitatory amino acid” was reported by Lambein et al. [19].

### 2.3. Interaction Between Environmental Factors Versus *β*-ODAP Content

Although its advantages are well recognized, the cultivation of grasspea has recently decreased because its seedlings and seeds contain the neurotoxic *β*-ODAP, which has been related to neurological disorders in both humans and animals [48]. *β*-ODAP production in this crop is reportedly associated with the total quantity of free nitrogenous chemicals available in the grasspea. Therefore, under field conditions, nitrogen and phosphate may be the two nutrients that have the greatest impact on the concentration of neurotoxins. In contrast, cyan alanine synthase was shown to be the primary enzyme for *β*-ODAP accumulation in grasspea, although the quantity of amino acids (such as serine) was proven to be inversely related to *β*-ODAP accumulation [49].

The production of *β*-ODAP may be significantly influenced by both environment and genetics [48]. Studies have revealed significant variations within the germplasm, ranging from 0.02% to 2.59% [51]. A multilocational study in the 2003 season by the Deber Zeit Agricultural Research Center (DZARC) in Ethiopia revealed that depending on the varying growing environment under low to high stress conditions, *β*-ODAP levels in the same cultivars can increase or quadruple. However, increased *β*-ODAP production in grasspea can be caused by drought, excess iron or cadmium, and a lack of zinc in the soil [47]. The specific physiological and molecular mechanisms underlying the biosynthesis of *β*-ODAP in grasspea are still unknown, despite research suggesting that abiotic stresses may generate an imbalance to alter the plant's osmotic potential and trigger *β*-ODAP formation [52].

During the different stages of growth, *β*-ODAP levels varied, with young leaves and mature seeds having the highest concentrations, 0.5%–2.5% and 0.5%–2%, respectively. Field research has shown that *β*-ODAP levels in seeds are positively linked to P and negatively correlated with Zn, N, Ca, K, or Mg in the soil [48]. It was also shown that *Rhizobium* infection definitely reduced the level of *β*-ODAP in seedlings. Stress from drought was also observed to increase the levels of *β*-ODAP in seedlings. As late planting is associated with low moisture stress, which exacerbates *β*-ODAP formation in the crop sink at the expense of output, early planting is favored for less toxicity.

Fertilization has been proposed to play a role in the *β*-ODAP concentration. Zinc treatment inhibited *β*-ODAP formation. When phosphorus and iron are present in abundance, they compete with zinc and increase its toxicity [3]. As a result, optimizing macro- and micronutrients produced optimistic results; grasspea has historically been the crop for harsh seasons, limited soil nutrients, and no inputs. By dispersing the entire production in the plant across and loads of seeds, growing the crop under better conditions can make it less hazardous and more productive.

It is believed that the neurotoxic component of grasspea operates as a zinc ion carrier molecule [53]. Soils poor in micronutrients due to monsoon rain floods, or otherwise deficient in accessible zinc and high iron content (Ethiopia vertisols), may be too accountable for the high amount of neurotoxicity in mature seeds, and therefore the high frequency of human lathyrism. This might explain why *β*-ODAP levels were higher in landraces from Bangladesh, Ethiopia, India, Nepal, and Pakistan than in those from North Africa, Turkey, Syria, and Cyprus. The absence of Zn in the soil is an agronomic concern in Bangladesh, particularly in monsoon-washed soils where grasspeas are grown in dry winters. Hence, a more balanced fertilization of the soil may lessen plant stress factors that cause toxicity, while boosting output.

Even among the poorest, occurrences of lathyrism under ordinary environmental conditions are almost expected [54]. For these reasons, the toxin levels released during stress situations are significantly more critical for crop protection than those under normal conditions. However, in highly bad circumstances, the variables above may combine to render even a generally low-toxin species unsafe to ingest in vast amounts. The development of cultivars that generate little or no toxin, even under harsh conditions, should continue to be a primary goal of breeding, and agricultural techniques that boost crop productivity appear to reduce agricultural toxicity, although more research is needed in this area. Since the threshold for human neurotoxicity in minimally nourished, poverty-stricken subjects is unknown, it is critically important to evaluate the safety of “low-ODAP/BOAA” strains of grasspea using appropriate animal species. Prolonged (at least 1-year long) feeding of three groups of minimally nourished nonhuman primates (preferably macaques) with “high,” “low,” and “zero ODAP/BOAA”, with periodic neurologic and terminal neuropathologic (brain, spinal cord) examination, is required to evaluate the safety of “low-toxin” strains. Therefore, the sensitivity of low *β*-ODAP production to the environment and the mechanism by which it is regulated deserve greater attention.

## 3. Current Opportunities and Challenges for Improving Grasspea Breeding and Molecular Tools

Grasspea cultivation is on the rise, with its expansion notably understated in official reports. Nevertheless, there is a widespread acknowledgment that South Asia and Ethiopia are witnessing an uptick in grasspea cultivation. Moreover, Europe shows promising potential for increased cultivation. Recent attention toward grasspea research is evident, with a notable surge in research efforts and momentum. This growing interest is further fueled by broader enthusiasm for new prebreeding techniques and climate-smart agricultural practices.

*β*-ODAP is the primary focus of grasspea literature, yet consumers and food processors believe that the narrative surrounding the risk of lathyrism has been exaggerated beyond its actual magnitude. Lathyrism incidents are infrequent, occurring under rare conditions, and despite the expansion of grasspea cultivation, reports of the disease remain scarce. The disproportionate emphasis on *β*-ODAP has hindered overall progress in grasspea breeding. Future initiatives should prioritize the development of safer grasspea varieties, integrated within broader breeding objectives aimed at enhancing yield and marketability.

Although uniting the grasspea community holds significance, it is crucial to recognize that diverse locations harbor distinct requirements for the crop. Disparities are evident both in research priorities and user demands. European researchers predominantly delve into fundamental research, aiming to comprehend the plant's genetics. Conversely, South Asian researchers concentrate on adaptive research and traditional breeding methods. Therefore, it is essential to acknowledge and accommodate these differences when fostering collaboration among grasspea researchers.

In recent years, grasspea (*Lathyrus sativus*) breeding perspectives have undergone a transformative shift, driven by the imperative to enhance both yield and safety. Breeders are now leveraging a combination of traditional and modern breeding techniques to develop cultivars with improved agronomic traits and reduced levels of *β*-ODAP, the neurotoxin associated with lathyrism. Through marker-assisted selection (MAS) and genomic selection, researchers can swiftly identify and introgress genes responsible for traits like disease resistance, drought tolerance, and higher yield potential. These advancements not only expedite the breeding process but also ensure the development of grasspea varieties that are safer for consumption, thus aligning with global food safety standards.

In parallel, the evolution of molecular tools has revolutionized grasspea research by providing unprecedented insights into its genetic makeup. With the advent of genome sequencing and bioinformatics, scientists can decipher the complex genetic architecture underlying desirable traits in grasspea. This knowledge not only expedites the breeding process but also facilitates the development of molecular markers for targeted trait selection. Moreover, genomic resources enable breeders to undertake precise genome-wide association studies (GWASs) to identify candidate genes associated with key agronomic traits, paving the way for more efficient and informed breeding strategies [19].

Simultaneously, advancements in food processing techniques have opened new avenues for enhancing the safety and nutritional quality of grasspea products. Researchers are exploring various detoxification methods such as soaking, boiling, fermentation, and enzymatic treatments to mitigate the neurotoxic effects of *β*-ODAP while preserving the nutritional integrity of grasspea seeds. By detoxifying grasspea seeds, these techniques not only ensure consumer safety but also unlock the crop's market potential, thereby offering farming communities an opportunity to increase income through value-added processing and diversified product portfolios. Ultimately, the convergence of breeding innovations, molecular tools, and food processing techniques holds immense promise for bolstering grasspea productivity and socioeconomic prosperity within farming communities, contributing significantly to income-doubling objectives and sustainable agricultural development [48].

### 3.1. Advanced Breeding Status to Overcome ODAP Content in Grasspea

To make grasspea safe for human consumption, efforts have been made globally to use these germplasms for the genetic improvement of grasspea with a limited molecular approach. In mapping studies, molecular markers have also been used to evaluate interspecific varieties and linkages in species evolution. Cross-amplified intron-targeted amplified polymorphisms, genomic simple sequence repeats, resistance gene analogs, and disease resistance indicators have all been successfully found in other legume species, but not in grasspea.

A few studies have also attempted to pinpoint the genes and underlying processes that grasspea use to impose biotic and abiotic stress or regulate the *β*-ODAP pathway. Proteomics has accelerated the identification of differential proteomics in response to salinity and low-temperature stress conditions, allowing researchers to quickly identify the shared signaling pathways involved in reducing abiotic stressors and identifying proteins that are differentially regulated [42]. The metabolic pathways associated with *β*-ODAP synthesis in grasspea were identified using metabolomics. To identify genes with unique functions, the grasspea genome is now being sequenced, which is essential for whole-genome resequencing and gene annotation. A draft grasspea genome sequence has recently been produced, and efforts are currently underway to resequence a variety of grasspea germplasm lines, totaling 384 germplasm lines [55].

The surprising observation that several agronomic traits of grasspea lines have low *β*-ODAP content raises the possibility that *β*-ODAP may be involved in plant growth and development [15]. Using traditional hybridization, mutant breeding, and somaclonal variation, several low *β*-ODAP lines have been created in Ethiopia and India [15]. As a consequence of advancements in grasspea breeding and various food processing procedures, new varieties of grasspea with subquantities (0.1%) of *β*-ODAP have been created. These novel versions have been shown to be safe for human consumption and do not induce lathyrism in individuals.

Current research on the toxicity of *β*-ODAP and the socioeconomic context of grasspea intake leads one to believe that in order to make grasspea safe, one should consider toxin levels under stress settings rather than under normal circumstances. This is influenced by the following predisposing factors.

I. All crops in a regional farming system are likely to be affected by stress events, notably drought and flooding, which might result in crop losses and subsequent scarcity of food and resources. Considering that grasspea can withstand stress conditions better than other food crops, this might result in a rise in grasspea consumption compared to other meals [54].
II. Impoverished households may consume less food overall and have a smaller variety of foods, which might contribute to malnutrition and increase their risk of developing lathyrism [56].
III. Under most stress conditions, grasspea *β*-ODAP levels increase [3, 57, 58], increasing toxin consumption once again.
IV. The effectiveness of conventional procedures for detoxifying grasspea for consumption may be hampered, particularly during dry spells, when the water and fuel needed for extensive steeping and boiling of grasspea seeds may not be available. Thus, people may resort to different, less secure techniques of food preparation as a result [59].

### 3.2. Proximate Composition and Mineral Content

In 100 g of edible whole grasspea seed, one can find 8.4 g of water, 1457 kJ (348 kcal), 27.4 g of protein, 1.1 g of fat, 59.8 g of carbohydrates, 7.3 g of fiber, 127 mg of calcium, 410 mg of phosphorus, and 10.0 mg of iron [60]. Grasspea contains very little tryptophan and methionine. Raw whole seeds have a starch content of 41% on a dry matter basis; these oval granules are typically 25 mm long and 17 mm wide. Grasspea hay contains 14.6% water, 9.9% protein, 1.9% fat, 36.5% fiber, 31.0% nitrogen-free extract, and 6.1% ash (Table 2). Seeds of cultivars containing up to 0.22 g *β*-ODAP per 100 g seed may be fed to developing chicks at a rate of 400 g grasspea seeds per kilogram feed without affecting weight gain, fat, protein digestibility, or both.

### 3.3. Amino Acid Content of Grasspea

Grasspeas are deficient in methionine and cysteine (sulfur-containing amino acids) but are rich in lysine and have high levels of polyunsaturated fatty acids [62]. Grasspea, like other pulse crops, is appreciated and farmed for its high protein content in the seeds, which are not only rich in crude protein but also in amino acids, allowing impoverished people in their primary production zones to have a complete diet [63].

For grasspea seeds that are in a favorable ratio for eating by humans and animals, the estimated total amino acids and fatty acids are 19.69–23.48 g/100 g and 58%–80% in the same order, respectively [51–53]. Similar to other legumes, grasspea seeds have a high lysine content (6.4–20.4 mg/kg) but a low content of sulfur-containing amino acids such as cysteine (3.8–4.3 mg/kg) and methionine (2.5–2.8 mg/kg) (Table 3).

### 3.4. Constraints (Antinutritional Content) in Grasspea Consumption

Grasspeas are an excellent source of essential minerals, complex carbons, and proteins in the body. In less-developed countries, it is still a staple food for the population. Additional factors affecting its consumption include its flavors, the presence of several antinutritional components, inadequate protein digestion, and discomfort caused by consuming these pulses in the intestines [64]. People's fear of eating grasspea, the lack of high-quality seeds with low levels of neurotoxins, and standardization of grasspea manufacturing technology are the three main issues.

Many chemical substances formed by secondary metabolism can be found in pulses. As these substances play no role in the direct metabolism of plants, they are not necessary for healthy growth and development. However, they support a variety of biological and ecological roles in plants and have a wide spectrum of biological activities. Antinutritional factors can block nutrients, restrict metabolism, or limit digestion, making them poisonous, disagreeable, or antinutritive for human ingestion [65].

Similar to other legumes, grasspea seeds contain compounds known as antinutritional factors, such as trypsin inhibitor [66]. This trypsin inhibitor, categorized under serine proteinase inhibitors (serpins), can impair the nutritional value of seeds by reducing protein digestibility and absorption, consequently hindering growth. Although traditionally viewed as detrimental, certain trypsin inhibitors have been discovered to offer benefits to both humans and animals, and some can even serve as agents in food processing [67]. Furthermore, trypsin inhibitor has been implicated in providing partial resistance against the bruchid or pulse beetle (*Callosobruchus chinensis*) across various legume species, including grasspea [68].

The antinutritional contents of different varieties of grasspea are presented in Table 4. Ethiopian grasspea *β*-ODAP ranges from 0.20% to 0.55% of the seed fresh weight [70]. Low-toxin grasspea varieties are grown elsewhere in the world, although they are still in the developmental phases of Ethiopia. Recently, the low-toxin cultivar *Wasie* was made available for extensive cultivation in Ethiopia [3]. They are considered safer to manufacture for ingestion when using thorough processing and combining processes with careful monitoring. However, researchers must evaluate and design the problems of environmental and outcrossing contamination (27%). The *β*-ODAP values of all the seed samples obtained from Fogera in Ethiopia range from 0.2% to 0.8%. The dehusked samples had values ranging from 0.2% to 0.7% (Table 4). The flour had a *β*-ODAP content of 0.15%–0.4%.

### 3.5. Contribution of Grasspea for Food Security and Protein Source

Exploiting underutilized plants is becoming more important because of major concerns about food security and agricultural sustainability, particularly owing to global climate change. Nutritionally dense grasspeas, which were previously underutilized and suitable for low-input agriculture and harsh settings, have received attention, and their significance is now widely acknowledged worldwide [15]. Although grasspeas are the least liked of the common edible legumes, their capacity to flourish in conditions such as drought and flooding makes them attractive to farmers with limited resources. Because of this, grasspea can be used as a last option if other crops have failed and do well in dry and poor soils. However, the presence of *β*-ODAP in seeds is a serious disadvantage because it jeopardizes the health of consumers. Thus, the creation of high-yielding, *β*-ODAP-free cultivars with low *β*-ODAP concentrations is a top priority in grasspea breeding. Additional studies are required to determine how to effectively detoxify foods without compromising their nutritional contents.

Grasspeas are the only readily available food source for the poorest segments of the population when other crops fail due to hardship; they can even be a survival meal during drought-induced famine. For low-income households, grasspeas are typically the least expensive edible legumes, making them a cornerstone of their traditional diet. Moreover, the seeds of this plant contain significant amounts of free l-homoarginine, a precursor of lysine in the human diet [71]. The use of grasspea grain has been constrained despite its high protein content owing to the presence of *β*-ODAP, a water-soluble nonprotein amino acid.

Grasspea is safe for consumption by humans when included in a healthy diet [72], and because seeds can partially detoxify through various processing techniques such as fermentation, presoaking in alkaline solutions, cooking, and other methods, it is now agreed that *β*-ODAP content in and of itself does not appear to be a problem [15]. Another suggestion is that nitriles, not *β*-ODAP, are the culprits behind lathyrisms [73]. In addition, the potential pharmacological benefits of *β*-ODAP should not be ruled out by any potential *β*-ODAP pharmacological benefits [44].

### 3.6. Food Processing Technologies and Grasspea

Legumes are extensively used in the food-processing industry. After being properly soaked and germinated, legumes have a much higher nutritional content and significantly less antinutritional material such as enzyme inhibitors, toxic amino acids, and other antinutrients. The chemical characteristics of the nonnutritive chemicals, the circumstances and intensity of the chosen processing technology, and the type and variety of the legume all affect how well the processing approach works. The nutritional content of grasspeas may change depending on how they are processed into various food and feed products. Even if the nutritional content of grasspea changes, the alterations that are known to occur during typical thermal processing do not have a noticeable harmful effect. Even if one method is more effective than another, any effort made to prepare grasspea food reduces toxicity [3]. Modern technology in breeding and food processing are the two primary strategies for reducing the concentration of *β*-ODAP to levels that are safe for human consumption.

### 3.7. Cooking Quality of Grasspea

The main disadvantage of using grasspea is that its seeds are difficult to rehydrate, to cook, to remove the seed coat, and to tenderize them; conventional processing methods need lengthy boiling times. The permeability of the seed coat and cotyledons to hot water had an impact on the amount of time it took to cook legumes, especially grasspea. Several studies have reported the impact of processing techniques such as blanching, soaking, and cooking on water absorption, leached solids, swelling power, cooking time, and sensory aspects of grasspea seeds. Blanched grasspea seeds were steeped for 12 h in each of the three soaking solutions, and the resulting reductions in cooking time were 60%, 73%, and 68%, respectively [74] (Table 5).

This is most likely the oldest method of making legumes palatable. Typically, it entails soaking the seeds first and then cooking them in boiling water until they become soft. Akalu, Johansson, and Nair [77] discovered a statistically significant reduction of 57% in grasspea flour after 60 min of pressure-cooking at 1500°C and a reduction of 39% in dry seeds after 30 min of autoclaving. Human participants who ate cooked grasspea seeds or a controlled dose of pure *β*-ODAP excreted less than 1% of it in their urine but increased their oxalic acid excretion. Higher quantities (between 21.1% and 75.2%) of orally or intraperitoneal administered radiolabeled *β*-ODAP were eliminated in the urine of mice, rats, and chicks [78]. The effects of cooking for 10, 20, and 30 min varied. Cooking for 20 min led to a significantly greater reduction in *β*-ODAP content than cooking for 10 or 30 min at all temperature intensities [79].

The following *β*-ODAP concentrations were discovered in various grasspea preparations in Ethiopian grasspea-based food items according to Asnake [3]. The computed percentage decrease in *β*-ODAP of the food items compared to raw *guaya* is shown in parentheses. The raw values were 480–780 mg/100 g, 228–412 mg/100 g (53–47%), 221–443 mg/100 g (54%–44%), and 72 mg/100 g (89%) in the gravy and cotyledon, respectively (*shiro-wot*) (Table 5). Unripe green peas or guayas are roasted or cooked. The results of a study conducted on the effects of various processed food products (*injera*, *shiro wett*, *kollo*, *kita*, and *nifiro*) consumed in Ethiopia, specifically in the Fogera District, Gondar administrative region in Shina district, revealed that the level of *β*-ODAP is highest in bread and roasted varieties of grasspea. When the steeping procedure was used, the level was lower in boiled snacks. The sauce made from the flour form *shiro* contained significantly lower amounts of *β*-ODAP and might not be as dangerous.

According to ethnographic research conducted in Ethiopia and India, the majority of consumers need to be made aware of the dangers of eating grasspea and adopt certain preparation methods to reduce toxicity. However, certain myths continue, such as the idea that steam created when cooking green peas is extremely poisonous [54]. However, no food preparation method can completely detoxify grasspea. Moreover, customers could be forced to use food preparation methods that are less successful in reducing toxicity if water or fuel is in short supply, as they commonly occur during droughts (e.g., by not discarding the water after boiling grasspea seeds).

#### 3.7.1. Soaking

Soaking is a component of several processes such as heating, canning, germination, and fermentation. This requires either abandoning the soaking medium or soaking the seeds in water, often until they reach their maximum weight. The type of legume, its species and variety, amount of time spent processing, temperature, pH of the soaking media, and storage conditions before processing all affect the results [80].

Soaking grasspea seeds before boiling can remove *β*-ODAP and reduce the risk of lathyrism by half [46]. Soaking (12 h) of cotyledon grits (1.5–2 mm) and roasting at 200°C for 37 min might be advised for a considerable decrease in *β*-ODAP, while enhancing starch in vitro digestibility. Similarly, Chatterjee et al. [81] discovered that soaking seeds in water for 7–8 h and then decanting the water eliminated the majority of *β*-ODAP. Moreover, they said that soaking split seeds in water overnight and decanting them enhanced the dal, making them nontoxic and suitable for consumption. *Dal* is a dish formed from pulses that have been split and stripped from their outer hulls (a staple of Indian, Pakistani, and Bangladeshi cuisine). When soaking mature grasspea seeds for 72 h in different soaking media, Malek, Sarwar, and Hassan [82] found an 11% and 13% increase in total protein and a 12% and 16% decrease in 1M NaOH and 0.1% wood ash solutions, respectively. They discovered that this process caused a decrease in total solids, nonprotein nitrogen, total soluble sugars, and reducing sugars.

Spending the night in lime water, toxins were eliminated after boiling. This drug also breaks down the trypsin inhibitors. No costly component is needed for processing grasspea seeds, because lime is continually available in most subcontinental homes for use with betel leaves [83]. Previous studies have shown that the toxic components of grasspea may be detoxified by water. To remove the toxin from grasspea seeds, Nguyen et al. [84] proposed two processing methods: steeping the dehusked seeds in hot water for several hours or boiling the seeds in water and draining away the supernatant. Both methods eliminated 70%–80% of the neurotoxins. However, mixing the dish with gravy containing antioxidant-rich seasonings decreases it by a factor of four, and the consumption of grasspea mixed with cereals high in sulfur amino acids was also very protective; however, the amount of the impact depended on the grasspea preparation ingested [46].

#### 3.7.2. Boiling

This process improves the appearance and sensory properties of the legumes. Boiling is generally performed for a few minutes at 100°C. To some extent, steaming or boiling techniques for preparation reduce toxicity by leaching the toxin out of the water while steeping or boiling. Boiling and fermentation with *Rhizopus oligosporus* and *Aspergillus oryzae* tend to be more effective in reducing toxicity than roasting, although they do not result in comprehensive detoxification [85–87]. *β*-ODAP levels were reduced by 90% by steeping, discarding the water, and then heating. Detoxification of grasspea through aqueous neurotoxic leaching is an important method of food preparation that reduces the risk of developing lathyrism. Several hours of steeping dehusked seeds in hot water and boiling the seeds in water removes 70%–80% of the harmful substances from the discarded supernatant. According to simulated kitchen tests, steeping grasspea in a significant volume of water for 3 min and then decanting the excess water eliminated approximately 30% of neurotoxic compounds [88].

### 3.8. Fermentation

Fermentation is the process of breaking down complex compounds into simpler ones using enzymes and bacteria. Fermented foods are more nutritious than their unfermented counterparts because of their improved protein quality, increased vitamin B synthesis, and reduced antinutritional factors. Chinese researchers discovered that turning grasspea into vermicelli may significantly reduce the quantity of *β*-ODAP to less than 1%. Furthermore, both microbial fermentation and two subsequent solid-state fermentations are effective in reducing neurotoxic *β*-ODAP [89]. Using solid-state fungal fermentation with the fungal strains *Rhizopus oligosporous* and *Aspergillus oryzae* in succession, the *β*-ODAP in grasspea was reduced by 80% on average for the high-toxin variety and up to 97% for the low-toxin variant [90]. Solid-state fermentation of several low-toxin genotypes of grasspea seeds with *Aspergillus oryzae* and *Rhizopus* microspores var. chinensis removed it from the seed meal [91].

#### 3.8.1. Extrusion

This approach broadens the product spectrum and enhances the consumption of the product matrix of leguminous seeds. Along with expanding applications, there has been increased interest in the physicochemical, functional, and nutritional effects of extrusion processing. When extrusion is applied explicitly to manufacture nutritionally balanced or fortified meals, such as weaning foods, dietetic foods, and meat replacers, nutritional concerns over extrusion cooking reach their pinnacle [92]. As a result, it significantly reduces the antinutrients in legumes [93]. *β*-ODAP levels in grasspea were reduced by 46% when extruded [85]. Antinutritional factors such as tannins were reduced by 77%, trypsin inhibitor was reduced to below detection limits, and *β*-ODAP was reduced by 46.9% after extrusion of grasspea seeds [94]. The enzymatic degradability of starch in grasspea increases from 11.8% in meal to 39.7% in extruded products [94]. Extrusion had no influence on phytate and nutritional components. Extrusion processing enhances the nutritional characteristics of grasspea by increasing growth, feed consumption efficiency, and *β*-ODAP in rohu fingerlings [95].

#### 3.8.2. Roasting

The process of cooking food uncovered in a metal frying pan is known as pan-broiling or roasting. The procedure has the advantage of reducing the moisture content of the food and thus improving its quality; nevertheless, nutritional losses, such as amino acids, might occur if the food becomes brown. It is also important to reduce and remove antinutritional factors. Roasted beans have distinct tastes, which may enhance their sensory appeal. Roasting also reduced neurotoxic concentrations in grasspea. As compared to intact raw seeds, Akalu et al. [96] reported a significant drop in *β*-ODAP of up to 30% after roasting milled samples and up to 67% after frying presoaked seeds. Girma, Tefera, and Dadi [54] discovered that different roasting, heating, autoclaving, and soaking combinations could reduce *β*-ODAP levels in entire seeds by up to 87%. There was also a pattern of increasing *β*-ODAP concentration decreasing with increasing temperature, with roasting resulting in a slight 20.61% decrease in the *β*-ODAP content [79]. According to Girma, Tefera, and Dadi [54], roasting and autoclaving milled grasspea generated considerable reductions in *β*-ODAP levels of up to 30% and 50%, respectively, when compared to raw whole seeds (Table 5).

### 3.9. Grasspea-Based Food Products

If the grasspea diet is processed and supplemented with adequate cereals or foods strong in sulfur-containing amino acids or antioxidants, such as onions, lathyrism can be prevented [97]. As a wider variety of food is available in urban settings than on subsistence farms that are vulnerable to drought, epidemics do not arise. Grasspea is once again highlighted as a regional and traditional product in some countries, including Italy; it is once more perceived as a costly and fashionable meal that people are ready to pay more than other pulse crops [98].

### 3.10. Grasspea Protein Isolate

In addition to their nutritional aspects, pulse proteins possess functional properties that improve their potential use in providing a wide variety of food products [99]. It is noteworthy that the functional properties of proteins can be altered by the protein source and procedures used for flour defatting and preparation, protein extraction procedures, and drying methods. Physiochemical parameters, such as pH, temperature, salt, and ionic strength, can also significantly affect the thermos and functional properties of proteins, as well as their structure [100]. Therefore, protein extraction from pulses has recently attracted the attention of researchers. The grasspea protein isolate mostly contains amino acid globulins (66%), glutens (15%), albumin (14%), and prolamin (5%) [101]. Moreover, the essential amino acids leucine, lysine, arginine, glutamic acid, and aspartic acid are all present in the grasspea protein.

Protein isolate powder drying may also result in the formation of irreversible insoluble clumps, but it enhances the stability and long-term storage of protein isolate powders. Different drying techniques can change the functional qualities of proteins because of their impact on the protein structure and denaturation [43].

### 3.11. Grasspea Starch

**Figure 1:**
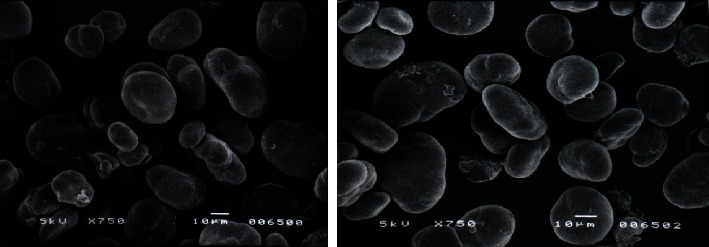
Microphotography of starch isolated from different grasspea varieties (http://www.ejpau.media.pl/volume4/issue2/food/art-10.html).

Due to their abundance in slowly digested starch and thus their low glycemic index, grasspea seeds have the particularly advantageous property of slowing down the digestion of starch. Because of its high amylose concentration and consequent vulnerability to retrogradation, grasspea starch may serve as a substitute for starches that have undergone chemical modification, and as a source of resistant starch (Figure 1). When both grasspea starches were pasted, the phase transition temperatures were greater than that of wheat starch but lower than that of regular corn starch. Although the paste viscosity of grasspea starch was much greater than that of wheat starch, the pasting temperature of the starch slurries was significantly lower for grasspea than for cereals. Grasspea starches exhibit much higher storage and loss moduli during the heating of starch suspensions.

### 3.12. Common Grasspea-Based Traditions in Food by Countries

The seeds are occasionally boiled in India; however, dal is the most common end product. In India, grasspeas are frequently used to make a variety of dishes, including *Khesari Dal*, which is typically served with rice and *chapatti* (Table 6). The production of *besan* also includes the use of grasspea, similar to other pulses (lentil, mung, and gram). By using innovative processing and value-adding techniques, farmers were able to produce food.

In a study by Malgorzata and Zbigniew [99], bread with 12% added grasspea whole meal was noted for its 14.51% protein content, 8.75% total dietary fiber content, and 3.90% and 2.89% soluble dietary fiber in water and acid solutions, respectively. It was already recorded in an Encyclopedia of Plants published in 1855 that bread prepared from a 50/50 blend of grasspea and wheat seemed to have no harmful effects; however, bread made solely from grasspea caused paralysis of the legs when consumed in continuation [3].

## 4. Conclusion and Remarks

Grasspeas are climate-resilient legume crops of critical socioeconomic significance, with approximately 290 million kilograms annually consumed as food in Ethiopia alone. Breeding initiatives should boost the content of sulfur-containing amino acids and antioxidants in grasspea, in addition to the usual attempts to reduce toxin levels. Consuming unprocessed grasspea should be avoided, especially when they are green, unripe, or boiled. Effective food preparation techniques are essential to reduce the neurotoxic hazards associated with eating grasspea. Even if the international community is unable to provide sufficient and timely food help, educational initiatives intended to increase awareness of these fundamental precautions are likely to reduce the risk of lathyrism. When legumes are properly soaked and germinated, their nutritional value increases dramatically, and the number of antinutritional substances, such as enzyme inhibitors, toxic amino acids, and other antinutrients, is drastically decreased. Soaking and boiling reduced the amount of *β*-ODAP in the seeds, and this effect was amplified if the water was changed after soaking and cooking. When seeds are ground into flour and used for baking or cooking, *β*-ODAP cannot be eliminated. Unripe green peas are dangerous and must be avoided at all costs. The consumption of roasted, boiled, and raw grasspea seeds should be minimized as much as possible. Educational and literacy initiatives must be implemented to address illiteracy rates. The illness has been linked specifically to the consumption of these seeds, rather than any other interactions with grasspea. Preprocessing techniques such as fermentation and prolonged soaking (> 18 h) have a significant impact, regardless of whether grasspea food processing features lower toxin levels to a certain extent. Eating gravy, unleavened bread, and fermented pancake did not increase the risk. Instead, it was linked to eating raw, unripe green grasspea and boiling grasspea. A correlational analysis revealed an inverse relationship between the number of new cases and the average quantity of food aid grains supplied per person. Before grasspea becomes the sole available food during famine, cereals and nutritional information should be made available to the populace. The full potential of this largely underused grain legume might be realized owing to the integrated, interdisciplinary approach now being applied to improve grasspea genetics and processing technologies.

## 5. Future Research and Outlooks

Particularly, in light of imminent climatic changes, grasspea variations make it an exceptional crop for ensuring nutritional security for resource-constrained farmers. However, its breeding and contemporary culture have been constrained by the presence of *β*-ODAP. Grasspea has great potential for use as a human food if the threat from *β*-ODAP can be eliminated. Although grasspeas have several distinctive qualities that make them appealing to growers and consumers, there is still more work to be done:

• As most people know nothing about grasspea, they unknowingly buy and consume grasspea seedlings in their daily lives. As a result, nutritional knowledge, education, and communication regarding healthy grasspea preparations are needed. Grasspea is a very effective nitrogen fixer and a highly regulated legume, and research into its nitrogen fixation mechanism will bring new insights into its cultivation. The role of *β*-ODAP in resistance to various abiotic stresses and nitrogen fixation in grasspea needs to be further explored.
• The effects of various processing treatments, such as soaking, germination, and cooking of germinated seeds, require further research on the *β*-ODAP content and other nutritional content of grasspea.
• Optimization of different food processing methods can produce low levels of *β*-ODAP content and has less effect on other important nutritional qualities.
• Moreover, the medicinal value of grasspea is also worthy to be further investigated.
• New grasspea genotypes that are safe for human consumption are needed from the breeding perspective, and the development of grasspea varieties with low seed antinutritional factor and *β*-ODAP content but with high seed protein content would be beneficial for human consumption, but before low-toxin strains are introduced, it is essential to prove their safety and nutritional viability experimentally in appropriate laboratory species, preferably in chronically fed nonhuman primates.

The integrated, interdisciplinary strategy presently used to enhance the genetics of grasspea and processing technology could allow the full potential of this mostly underutilized grain legume to be realized. We believe that addressing the aforementioned research objectives will help not only increase grasspea output but also introduce this crop to areas where it is presently not farmed.

## Figures and Tables

**Table 1:** Ethiopia's production of major legume crops (‘000 metric tons).

**Crop**	**Year**			
	**2013/2014**	**2014/2015**	**2015/2016**	**2016/2017**
Faba beans	992	839	849	855
Red kidney beans	259	312	380	355
Field peas	380	343	323	347
Chickpeas	424	459	473	343
Grasspea	317	251	288	239
Lentils	159	137	134	172
White pea beans	199	202	160	123
Other grain legumes	69	57	82	109
Total	2798	2600	2688	2542

**Table 2:** Proximate compositions and selected mineral content of some grasspea forms and genotypes of different ecolocations.

**Grasspea varieties or collection areas**	**Crude protein**	**Ash (%)**	**Moisture (%)**	**Crude fiber (%)**	**Crude fat (%)**	**Carbohydrate**	**Gross energy**	**Fe**	**Zn**	**Ca**	**Reference**
Grasspea; flour	22.10 g	1.7 g	9.30	2.4 g	0.9 g	66.00 g	360.50 ca	4.2 mg			[61]
Grasspea; sauce	2.3	1.00	79.60	0.90	9.30	7.8	124.1	1.6			
Grasspea; dried	22.60	4.30	10.70	8.20	1.40	61.00	347.00	10.82			
Grasspea; boiled	10.50	0.90	55.30	3.80	0.50	32.80	177.7	4.7			
46030	26.4	1.5	8.7	7.3	1.3	57.8	348.5	8.3		178	

46050	24.1	1.6	7.1	7.5	1.2	52.4	316.8	6.6		134	[60]
46052	25	1.8	8.5	7.5	1.2	55.0	330.8	11.1		133	
46053	28.1	1.9	9.6	7.2	1.2	52.7	334	10.7		154	
46054a	27.2	2.0	8.1	8.0	1.6	52.4	332.8	18.4		179	
46054b	25.7	1.5	8.9	6.8	1.4	54.6	333.8	7		177	
46058	24.9	1.9	8.9	7.2	1.5	59.6	351.5	8.2		177	
46060	24.4	1.8	8.2	7.2	1.2	56.4	334	8		178	
46070	25.0	1.8	8.5	7.7	1.1	56.4	331.5	7.7		156	
46071	25.6	2.1	7.5	8.1	1.3	55.0	329.9	7.5		132	
46072	25.4	1.8	8.6	7.7	1.3	54.6	332.5	7.3		132	
46073	24.6	1.8	8.5	7.7	1.0	56.4	338.9	6.9		133	
46074	26.3	2.1	7.6	8.3	1.3	54.0	323.4	7		133	
46075	24.9	1.9	8.9	7.2	1.5	55.1	337.3	9.4		178	
46076	24.8	1.9	7.4	8.1	1.4	55.8	336.3	7.2		134	
46078	24.0	1.8	8.5	7.7	1.1	54.6	330.2	7.4		178	
46080	24.2	1.8	7.7	7.2	1.2	57.1	336	16.9		177	
201513	25.7	1.7	8.3	7.2	1.2	55.1	334	11		134	
201538	24.1	1.6	7.1	7.5	1.2	57.4	336.8	18.1		176	
201540	25.4	1.5	7.4	7.7	1.0	55.6	333	11.4		176	
201543	25.3	1.5	7.3	7.7	1.2	55.8	335.2	6.9		132	
201545	22.6	2.1	8.9	7.8	1.3	57.1	330.5	8		200	
201547a	22.5	1.5	7.6	7.3	1.4	57.7	345.4	6.7		176	
201547b	23.6	1.6	7.3	7.7	1.2	57.8	336.3	13.2		79	
201547c	24.2	1.8	7.4	7.6	1.1	57.0	334.7	7.8		198	
Raw gray-mottled seed	22.81	2.75	8.26	8.57	0.61	65.26	357.77	4.84	3.00	242.76	

South Wollo-Gerado	28.17%	1.32		5.31	1.42	63.78	380.59	5.92	3.71	99.64	[46]
South Wollo-Abasoqotu	29.38	1.28		4.64	1.47	63.23	381.67	4.69	3.63	110.15	[60]
South Wollo-Assigido	27.82	2.33		7.41	1.29	61.15	367.49	5.88	3.01	94.33	
North Wollo-Gubalafto	28.07	1.87		3.56	1.33	65.17	384.93	5.25	3.11	100.95	
North Wollo-Habro	30.23	3.63		6.23	0.93	58.98	365.31	6.94	3.74	116.97	
Central Tigray-Axum	31.03	3.17		8.62	1.38	55.80	359.74	5.88	4.52	87.84	
North Gonder-Dembia	28.51	2.91		4.19	1.29	55.60	360.05	6.21	2.74	84.21	
North Gonder-Gonder Zuria	29.92	3.17		4.75	0.97	61.19	367.73	5.45	2.88	112.36	
East Gojam-Adet	31.18	1.82		6.83	0.98	59.28	369.81	5.28	3.60	87.01	
East Gojam-Bahir Dar Zuria	29.08	4.14		5.33	0.92	60.53	366.72	5.37	3.93	87.00	
North Shoa-Girar Jarso	27.29	3.87		7.24	1.13	60.47	361.21	4.64	3.01	89.90	
West Shoa-Wolonkomi	30.39	2.33		6.47	1.27	60.79	347.47	6.84	3.76	90.26	
Wes Shoa-Becho	29.93	1.95		5.22	1.23	61.67	372.47	8.74	3.43	82.01	
East Shoa-Gimbichu	31.14	2.87		7.15	1.17	57.67	365.74	6.33	3.32	98.89	
East Shoa-Akaki	31.98	3.18		8.48	1.17	55.05	359.91	5.03	3.62	94.13	

**Table 3:** Composition of essential amino acids in grasspea seed (based on 16 g of nitrogen) and estimates of amino acid requirements for adult humans (milligram/kilogram per day).

**Essential amino acids**	**Composition of amino acid in grasspea**	**Amino acid requirement for adult human**	**% of fulfilment based on WHO standards**
Histidine	2.51	8.12	30.9
Leucine	6.57	10	65.7
Isoleucine	6.59	14	47.1
Lysine	6.94	12	57.8
Methionine	0.38	13	2.9
Phenylalanine	4.14	14	29.6
Threonine	2.34	7	33.4
Tryptophan	0.40	3.5	11.4
Valine	4.68	10	46.8

*Note:* Source: http://www.fao.org/docrep/003/AA040E/AA040E05.htm#ch5.6.

**Table 4:** Antinutritional content of some grasspea varieties in mg/100 g.

**Samples from different areas of Ethiopia**	**Phytate**	**Tannin**	***β*-ODAP**	**Reference**
46030	878	710	263	[69]
46050	1028	600	231	
46052	952	658	214	
46053	715	856	271	
46054a	735	776	254	
46054b	992	675	193	
46058	768	585	281	
46060	863	585	273	
46070	578	675	294	
46071	798	500	291	
46072	983	600	281	
46073	875	700	282	
46074	858	675	254	
46075	732	765	253	
46076	622	520	293	
46078	677	717	314	
46080	640	820	272	
201513	942	767	314	
201538	817	780	194	
201540	678	850	223	
201543	525	800	221	
201545	625	670	297	
201547a	642	558	172	
201547b	538	618	253	
201547c	797	820	252	

Raw gray-mottled seed	352.04	579.76	421.18	[46]

South Wollo-Gerado	693.19	578.64	674.21	[60]
South Wollo-Abasoqotu	883.47	734.27	836.14	
South Wollo-Assigido	547.63	724.48	518.29	
North Wollo-Gubalafto	584.51	719.39	638.38	
North Wollo-Habro	1041.37	452.53	831.23	
Central Tigray-Axum	994.73	756.67	891.31	
North Gonder-Dembia	558.42	866.29	636.27	
North Gonder-Gonder Zuria	712.87	596.73	782.34	
East Gojam-Adet	894.41	784.62	830.41	
East Gojam-Bahir dar Zuria	669.92	714.23	698.29	
North Shoa-Girar Jarso	635.39	762.81	587.15	
West Shoa-Wolonkomi	823.35	742.95	737.27	
Wes Shoa-Becho	717.57	776.78	756.37	
East Shoa-Gimbichu	1098.62	458.25	821.45	
East Shoa-Akaki	1008.89	518.31	1001.49	

Somaclonal line				
ILAT-LS-K-290			168	[14]
ILAT-LS-K-30			216	
ILAT-LS-K-104			140	
ILAT-LS-K-33			125	
ILAT-LS-K-299			206	
ILAT-LS-K-289			104	
ILAT-LS-K-444			104	
ILAT-LS-K-387			211	
ILAT-LS-K-190			202	
ILAT-LS-K-288			119	
ILAT-LS-K-390			168	
Local variety			259	

**Table 5:** The effects of different processing methods on the nutritional and antinutritional content of grasspea.

**Processing methods**	**Crude protein (%)**	**Ash (%)**	**Moisture (%)**	**Crude fiber (%)**	**Crude fat (%)**	**Carbohydrate (%)**	**Gross energy (kcal/100** g**)**	**Fe (mg/100 g)**	**Zn (mg/100 g)**	**Ca (mg/100 g)**	**Tannin (mg/100 g)**	**Phytate (mg/100 g)**	***β*-ODAP (mg/100 g)**	**References**
Raw gray-mottled seed	22.81	2.75	8.26	8.57	0.61	65.26	357.77	4.84	3.00	242.76	352.04	579.76	421.18	[75]
Wet roasted	24.97	2.52	5.69	8.61	1.17	62.73	361.33	4.94	3.09	254.40	235.14	266.37	370.50	
Boiled	25.18	2.30	7.30	8.55	1.01	62.97	361.58	4.60	2.93	301.34	247.26		260.0	
Unleavened bread (*kitta*)	21.39	2.78	4.16	3.31	0.95	71.57	380.37	5.34	3.33	254.40	102.16	48.30	431.58	
*Shiro-wot*	20.88	2.34	7.18	6.20	0.60	69.98	368.82	4.71	3.06	207.94	90.40	97.92	222.3	

Raw local variety											670	467.3	476.3	[76]
Autoclaving											533	368.2	431.1	
Dry heating											244	NIL	120	
Boiling											438	462.3	422.0	
Cooking											178	190.0	111.0	
Dehulled seeds											2.01	467.0	476.3	
Boiled 15 m											0.90	408.9	125.7	
Soaked 18 h											0.62	350.0	90.0	
Boiled 30 m											0.31	256.0	54.3	
Tempeh fermentation											13.27	7.5	35.3	

Raw grasspea	26.02							60.7	43.85	1283	[22]			
Roasting	26.60							61.4	48.2	1318				
Soaking	27.60							62.2	43.6	1568				
Soaking followed roasting	27.87							63.0	45.3	1537				
Germination	28.70							62.0	47.8	2146				
Germination followed roasting	27.3							66.6	80.4	2270				

Soaking in acidified water in different hours														
0	25.6	2.8		7	0.8	53.6	[74]							
1	26.9	2.7		7	0.8	53.6								
3	27.4	2.6		7.1	0.8	52.1								
6	27.7	2		7.3	0.9	52.1								
18	28.0	1.7		7.4	1	51.6								
24	28.5	1.5		7.6	1.1	51.3								
48	28.6	1.5		7.8	0.8	51.2								
78	28.6	1.4		8	0.9	51.1								
Tap water in different hours														
0	25.6	2.8		7	0.8	53.9								
1	25.8	2.7		7.1	0.8	53.6								
3	26.0	2.6		7.2	0.8	53.4								
6	27.2	2.3		8.8	0.9	50.8								
18	27.5	2.3		9	1	50.2								
24	27.7	2.4		9.4	1.1	49.4								
48	28.0	2.4		9.4	1	49.2								
78	28.3	2.3		9.4	0.9	49.1								
NaOH solution in different hours														
0	25.6	2.7		7	0.8	53.9								
1	25.4	2.7		7	0.8	54.1								
3	25.1	2.5		7.1	0.8	52.5								
6	24.6	2.3		7.3	0.9	53.4								
18	24.1	1.8		7.8	1	53.3								
24	23.6	1.9		7.6	1	52.9								
48	23.1	2		7.9	1.1	52.9								
78	22.6	2.1		9.2	1.1	53.0								
Wood ash filtrate in different hours														
0	25.6	2.8		7	0.8	53.9								
1	25.6	2.8		7.2	0.8	53.6								
3	25.5	2.9		8	0.8	52.8								
6	25.1	3.1		8.8	0.9	52.1								
18	24.8	4.7		9	1	51.1								
24	24.3	4.7		9.4	0.9	50.2								
48	23.7	4.7		9.4	0.8	51.4								
78	21.5	4.7		9.4	0.8	51.8								

**Table 6:** Different traditional grasspea-based food products and their preparation mechanisms for utilization.

**No.**	**Product name**	**Characteristics, processing conditions, and mode of utilization of chickpea-based food products**	**Countries**
1	Gahana Bari	Grasspea (50%) and black gram (50%) or grasspea alone (100%). The water must be emptied after soaking the grasspea and black gram in water for a whole night. The black gram and grasspea are then ground separately in a mixed grinder. Equal portions of black gram dust and grasspea are blended. Because sesame is used as the primary ingredient, Gahana Bari can be consumed up to 4 months after it is made. Because poppy seed is one of the primary components, Gahana Bari can be consumed without risk 10 months after it is manufactured [102].	India
2	Phul Bari	The main component grasspea is steeped in water for the whole night. The seed is thoroughly ground using a combination grinder once the water has been drained. The ingredients are thoroughly blended while maintaining the proper moisture level after the addition of salt, fennel seed, ginger, and dried chilies in the exact quantities. When cooked in a frying pan at a low temperature, the meal takes 2–3 min to prepare. It goes nicely with rice and chapatti and may also be eaten as a snack or an appetizer with tea.	India
3	Extruded product	Cereal-based extruded products are particularly popular snacks, especially during tea breaks and when travelling. The delicious extruded products also provide better nutritional value. Gluten-sensitive people experience problems while eating Kurkure produced from wheat, but not when eating Kurkure made from grasspea flour. Using a combination of components, such as maize flour (70%), rice flour (10%), and grasspea flour (20%), the extradites are created [102].	India, Japan, and China
4	Roti	Grasspea flour is a staple dish for landless peasants in Bangladesh. In Asia, immature pods are either cooked and consumed as a vegetable or boiled, salted, and consumed as a snack. In addition to being cooked as green vegetables, young vegetative sections are dried and utilized as vegetables during the off-season. In India, Bangladesh, and Pakistan, green pods and seeds of a leafy vegetable are consumed raw or fried with salt as snacks. Young vegetative parts (4–6 cm) are picked and cooked similarly to green vegetables.	Bangladesh and Indian
5	Grasspea tempeh	It is a traditional Indonesian dish. With the use of controlled fermentation and natural culturing, soybeans are bonded into a cake-like structure. *Rhizopus oligosporus* or *Rhizopus oryzae*, sometimes referred to as tempeh starter, is the fungus used in the fermentation process. Pretreatment green peas were inoculated with a powdered inoculum of *Rhizopus oligosporus* at a density of 10 colony-forming units per gram (cfu/g) of a prepared substrate in order to manufacture tempeh.	Indonesia
6	Miso	It is a Japanese fermented paste that is added to soups or used as a garnish. Miso's ingredients included soybean or grasspea, unpasteurized miso, sea salt, and demineralized water (both salty and sweet) (https://www.epicurious.com/ingredients/how-to-buy-store-and-cook-with-miso-paste-recipes-article). Throughout the fermentation process, the ascorbic acid and phenolic component levels of all miso increased, but the miso prepared with grass-fed peas had the highest nutritious value. The fastest growth and darkest color were likewise displayed by grasspea sweet miso. Two textural parameters, firmness and adhesiveness, decreased with time as miso made from brass pea and soybean was stored, with grasspea sweet miso showing the greatest firmness drop (51.63 N–6.52 N) and soybean sweet miso having the greatest adhesiveness reduction (27.76 N–3.11 N). The textural characteristics and viscoelastic moduli of the miso made from grasspea were comparable to those of the control miso manufactured from soybean after 4 months, demonstrating that grasspea may be utilized as a raw material to create a sustainable miso with potential health benefits [103].	Japan
7	Mixed milk	To prepare it for eating, Chinese people currently bake the seeds, stir-fry, or boil the sprouts in broth with other foods like eggs, meat, shrimp, or other vegetables. Various pretreatments were employed to detoxify and dehull grains in order to inactivate lipoxygenase and improve milk taste. These pretreatments included heating, ethyl alcohol immersion, and pH adjustment. In terms of all the sensory assessment aspects, the results revealed that the milk sample comprising 5% skim milk powder, 5% grasspea milk, 3% sugar, and 1.68% fat was regarded to be the best. The best outcomes were obtained using a mixture of grasspea milk and skim milk powder at a 50 : 50 ratio for total solids [104]. Broadly speaking, as shown below, the Ethiopian “culinary pattern” involves the usage of grasspea in four various forms: soups (or soup-like foods), sauces, processed seeds, and unleavened bread [100].	
8	*Eshet* (unripe green)	Grasspea is collected from farmsteads during the harvest season and directly consumed as a snack without preparation.	Ethiopia
9	*Kollo* (roasted)	Grasspea is briefly soaked in boiling or cold water, after which excess water is decanted and the seed is roasted and consumed as it is.	Ethiopia
10	*Nifiro* (boiled)	Grasspea is washed with hot or cold water two or three times; then, cold water is added, and the pulse is cooked until it is soft enough to be eaten.	Ethiopia
11	*Kitta* (bread)	The grasspea is husked and crushed into flour, potentially with cereals of varied types and amounts; the flour is then combined with water, and a thick batter is formed and baked into unleavened brea.d	Ethiopia
12	*Injera* (pancake)	The grasspea is husked and crushed into flour, potentially with cereals of varied types and amounts; after combining the flour with yeast and water, a fairly thin batter is created, which is stored at room temperature until fermented. *Injera* is made from fermented dough (pancake).	Ethiopia
13	*Shiro* (gravy)	The grasspea is softly roasted, then thoroughly cleaned in cold water and lightly toasted once more before being husked and milled into the flour that is needed to make the Ethiopian gravy “*Shiro-wot*.” The gravy is only eaten with *Kitta* or *Injera*.	Ethiopia
14	*kik wott*	(Sauce made of hulled split seeds) is eaten together with “*injera*” (a pancake-like unleavened bread).	Ethiopia

## Data Availability

All the data used in this study is included in the manuscript itself.
